# Supplementary material for: STRA8–RB interaction is required for timely entry of meiosis in mouse female germ cells
Source: Nat Commun. 2023 Oct 25;14:6443. doi: 10.1038/s41467-023-42259-6 (PMC10600341; doi:10.1038/s41467-023-42259-6)
Supplement: Supplementary file 1 — Supplementary Information [file 41467_2023_42259_MOESM1_ESM.pdf]

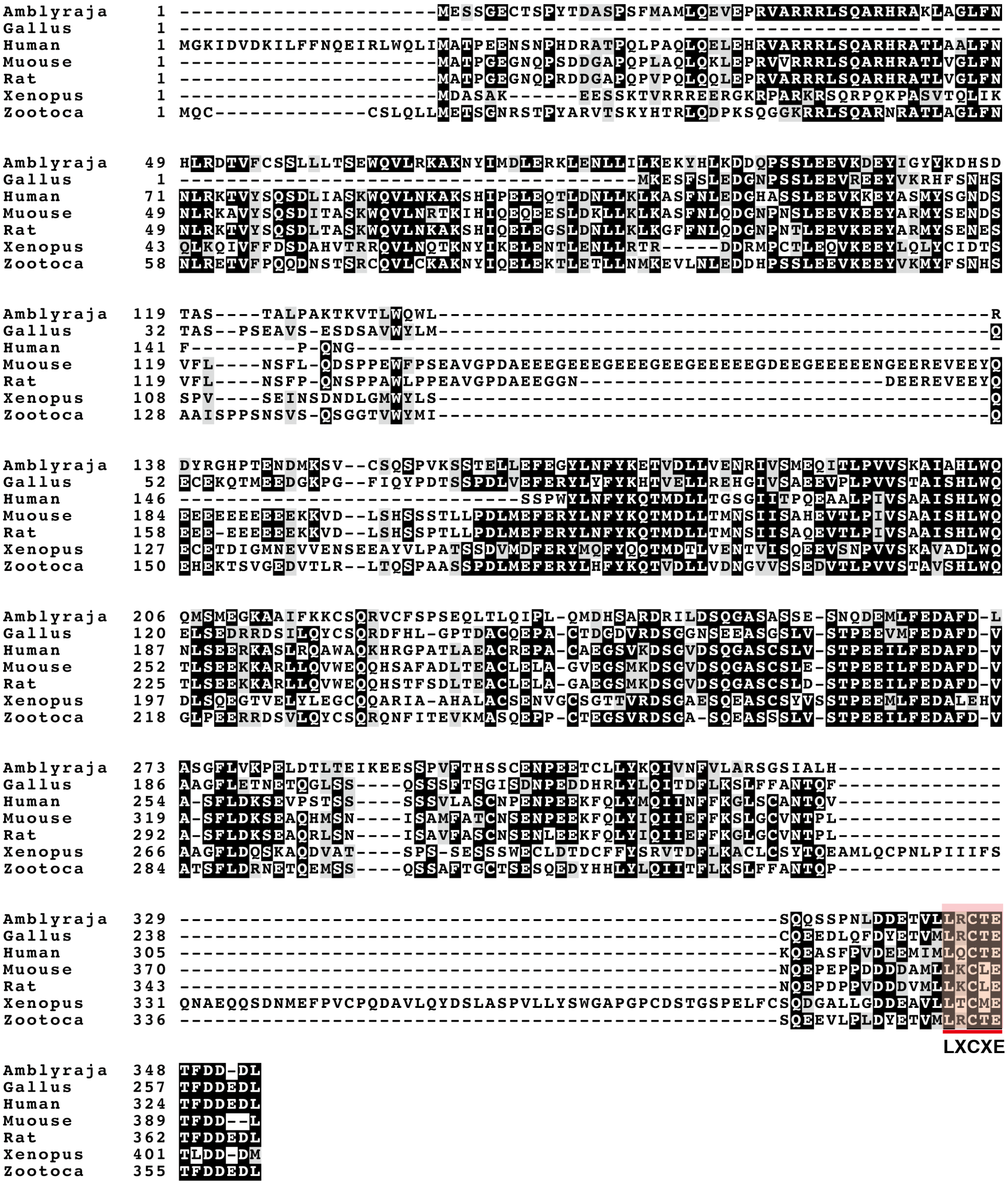

Supplementary Figure 1. LXCXE motif of STRA8 is conserved among vertebrates.

The amino acid sequences of STRA8 homologs of *M. musculus* (mouse, NP_033318.1), *R. norvegicus* (rat, XP_006236344.1), *H. sapiens* (human, NP_872295.2), *Xenopus tropicalis* (amphibia, XP_031753974.1), *Gallus gallus* (chick, XP_015145052.1), *Zootoca vivipara* (reptile, XP_034984057.1) and *Amblyraja radiata* (fish, XP_032894256.1) were obtained from the National Center for Biotechnology Information-National Institutes of Health (NCBI-NIH) protein database. The LXCXE motif of STRA8 is highlighted in red.

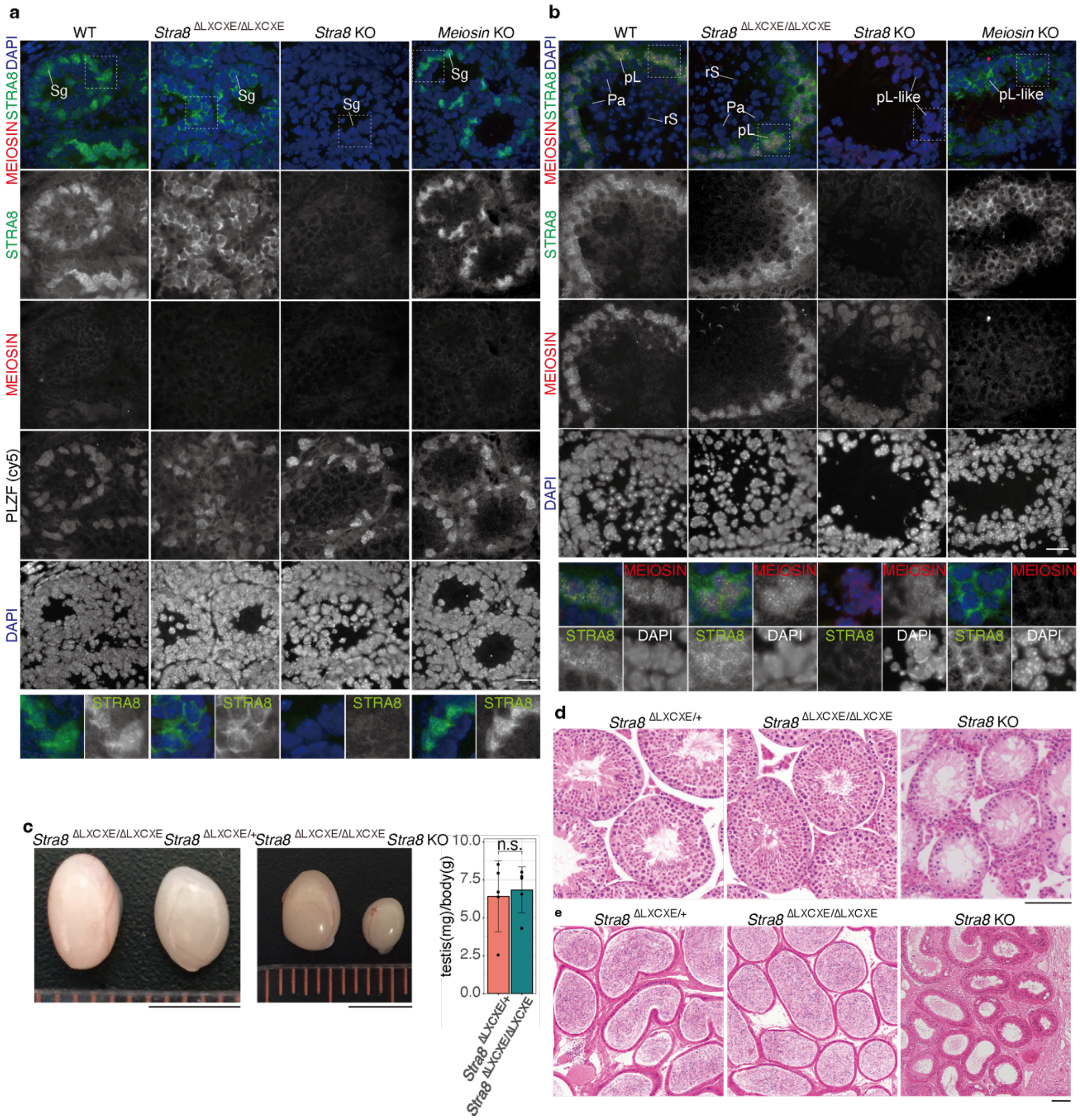

Supplementary Figure 2. Analyses of *Stra8* LXCXE mutant male germ cells. (Related to Figure 1)

(a) Seminiferous tubule sections from wild type (WT), *Stra8*$^{\Delta\mathrm{LXCXE}}$-*3FH* KI homozygous, *Stra8* KO and *Meiosin* KO testes were immune-stained. To facilitate the observation of STRA8 positive differentiating spermatogonia, neonatal male mice were subjected to consecutive injection of a RA synthesis inhibitor WIN 18,446 from P2 to P6 to block spermatogonial differentiation, followed by RA injection at P7. Seminiferous tubule sections at P8 were stained for MEIOSIN, STRA8, PLZF (Cy5) and DAPI. Sg: spermatogonia. Scale bar: 25 µm. Enlarged images are shown on the bottom.

(b) Seminiferous tubule sections from WT, *Stra8*$^{\Delta\mathrm{LXCXE}}$-*3FH* KI homozygous, *Stra8* KO and *Meiosin* KO testes (eight-week-old) were stained as indicated. pL: pre-leptotene spermatocyte, Pa: Pachytene spermatocyte, rS: round spermatid. Scale bar: 25 µm. Enlarged images are shown on the bottom.

(c) Testes from *Stra8*$^{\Delta\mathrm{LXCXE}}$-*3FH* KI homozygous and heterozygous, and *Stra8* KO (four-week-old). Scale bar: 5 mm. Testis/body-weight ratio (mg/g) of *Stra8*$^{\Delta\mathrm{LXCXE}}$-*3FH* KI homozygous and heterozygous mice (n = 5, respectively) is shown on the right (mean with SD). There was no statistical significance between the two. n.s.: not significant; $p = 0.7418$ (Welch's t-test).

(d) Hematoxylin and eosin staining of the sections from *Stra8*$^{\Delta LXCXE}$-*3FH* KI heterozygous and homozygous and *Stra8* KO testes (eight-week-old). Scale bar: 100 μm.
(e) Hematoxylin and eosin staining of the sections from *Stra8*$^{\Delta LXCXE}$-*3FH* KI heterozygous, *Stra8*$^{\Delta LXCXE}$-*3FH* KI homozygous and *Stra8* KO epididymis (eight-week-old). Scale bar: 100 μm.

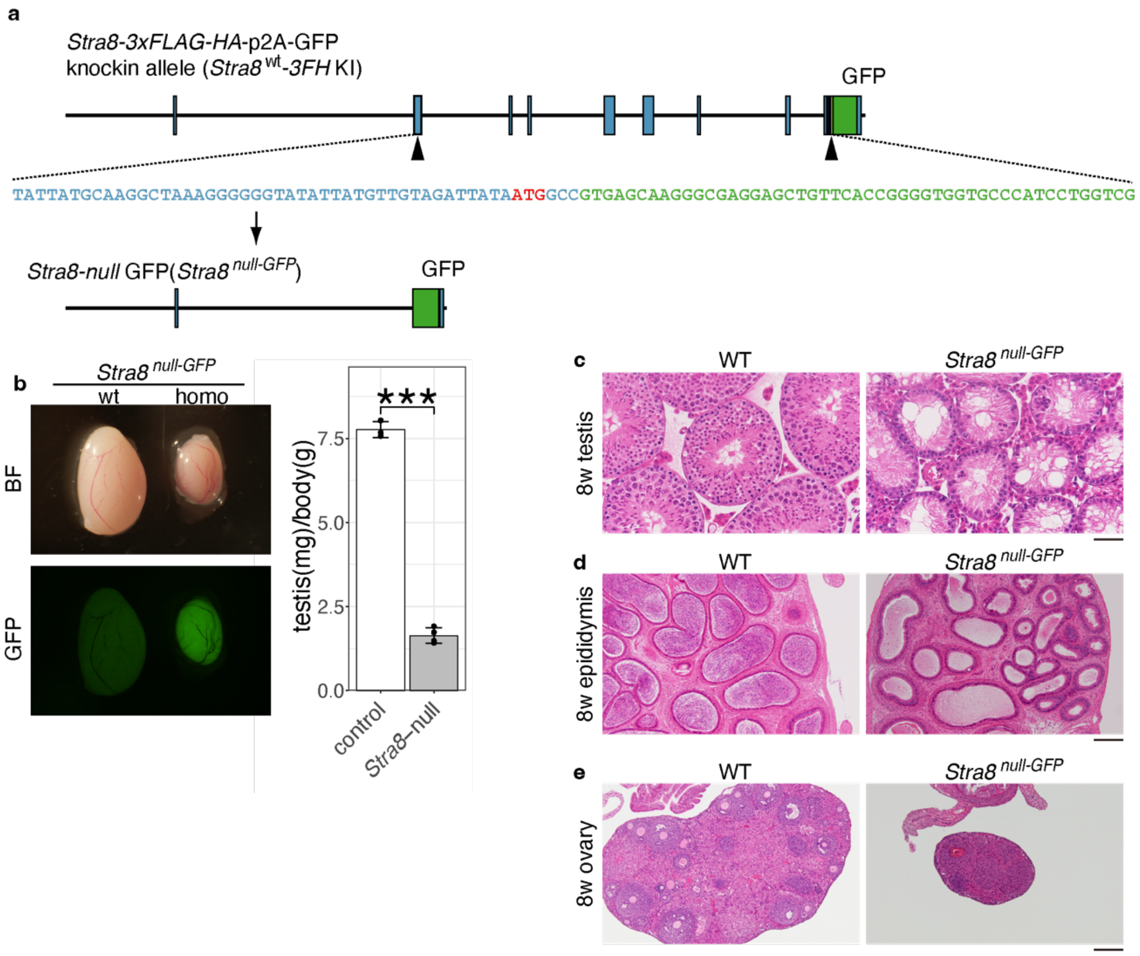

Supplementary Figure 3. The phenotypic evaluation of *Stra8^{null}-GFP* mouse. (Related to Figure 2)

(a) Schematic illustrations of the *Stra8-3xFLAG-HA-p2A-GFP* KI allele (*Stra8*^{wt}*-3FH* KI) and the *Stra8*-null *GFP* knock-in (*Stra8*$^{null\ GFP}$-KI) allele. *Stra8*$^{null\ GFP}$-KI allele was generated by removing all the *Stra8*-coding exons (Exon2-Exon9) from *Stra8*^{wt}*-3xFLAG-HA-p2A-GFP* knock-in allele. Blue boxes represent exons. Coding exon 9 was followed by *3xFLAG-HA-P2A-GFP* and the 3'UTR. Triangle: CRISPR gRNA target sites. ssODN sequence is shown. The start codon and *Gfp* sequences are shown in red and green, respectively.

(b) Testes from *Stra8*$^{null\ GFP}$-KI homozygous and control wild type males (8-weeks old). Bright field (upper) and fluorescent GFP (lower) images are shown. Testis/body-weight ratio (mg/g) of *Stra8*$^{null\ GFP}$-KI homozygous and control heterozygous/wild type mice (8-weeks old, n=3 for each genotype) are shown on the right (mean with SD). Statistical significance is shown by ***: $p < 0.0001$ (Two-tailed t-test).

(c) Hematoxylin and eosin staining of the sections from WT and *Stra8*^{null}*-GFP* homozygous testes (eight-week-old). Scale bar: 50 μm

(d) Hematoxylin and eosin staining of the sections from WT and *Stra8*^{null}*-GFP* homozygous epididymis (eight-week-old). Scale bar: 200 μm

(e) Hematoxylin and eosin staining of the sections from WT and *Stra8*^{null}*-GFP* homozygous ovaries (eight-week-old). Scale bar: 200 μm

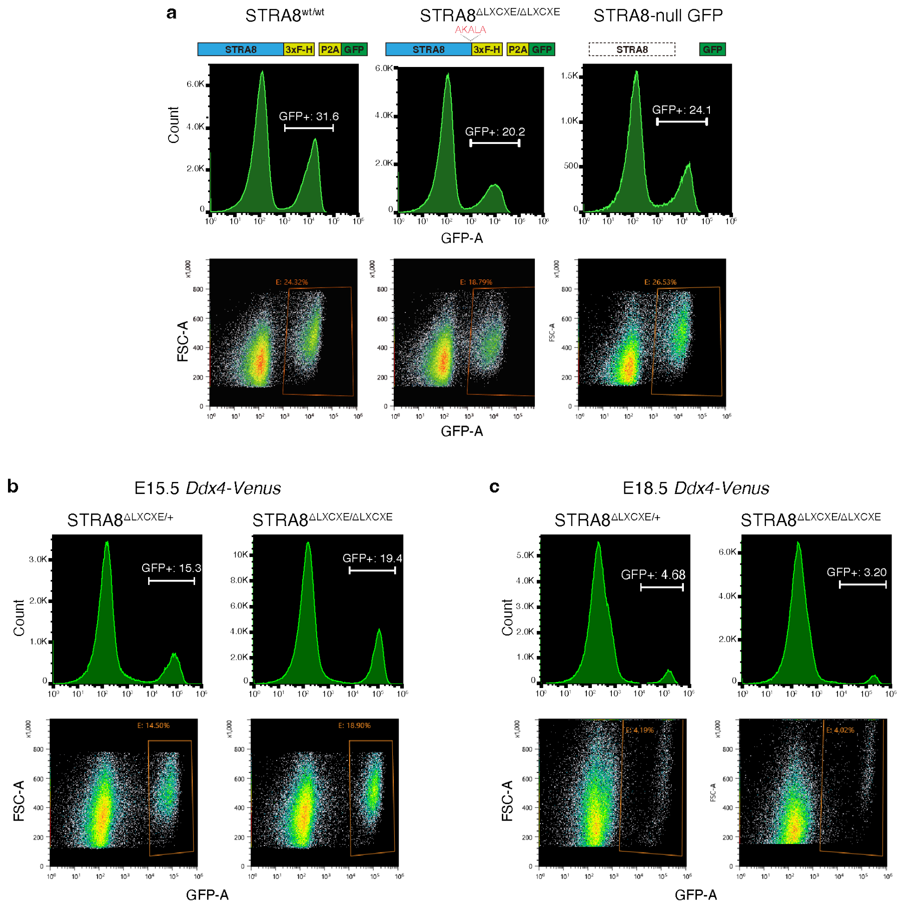

d

Platform: Chromium (10x Genomics)

Sampling stage:	E14.5			E15.5		E18.5	
Genotype:	*Stra8* wt 3FH-KI/wt 3FH-KI	*Stra8* ΔLXCXE/ΔLXCXE	*Stra8* null GFP/ null GFP	*Stra8* ΔLXCXE/+	*Stra8* ΔLXCXE/ΔLXCXE	*Stra8* ΔLXCXE/+	*Stra8* ΔLXCXE/ΔLXCXE
Cell type:	STRA8-expressing female germ cell			DDX4-expressing female germ cell		DDX4-expressing female germ cell	
Number of used embryos:	12	7	3	4	10	9	8
Total number of genes detected:	20,747	21,021	22,083	20,802	20,802	22,169	23,722
Sequencing Saturation:	82.9%	79.2%	75.9%	67.4%	73.8%	78.3%	81.3%
Mean reads / cell:	499,429	275,422	300,919	186,276	259,778	245,794	261,605
Median genes / cell:	7,396	6,715	7,364	6,038	6,468	5,574	5,256
Median UMI counts / cell:	53,769	38,640	49,068	35,134	38,095	25,422	21,780
Total number of cells:	891	1,562	1,415	2,070	1,537	1,516	1,396
Total number of cells after QC:	734 (82.4%)	1,393 (89.2%)	1,190 (84.1%)	1,830 (88.4%)	1,353 (88.0%)	1,070 (70.6%)	938 (67.2%)

Supplementary Figure 4. scRNA-seq analyses of STRA8-expressing germ cells isolated from *Stra8* $^{wt/wt}$, *Stra8* $^{\Delta LXCXE}$ and *Stra8* ^{null} embryonic ovaries. (Related to Figures 2, 5 and 6)
(a) The GFP positive cells were isolated from *Stra8* ^{wt}*-3FH-p2A-GFP* KI, *Stra8* $^{\Delta LXCXE}$*-3FH-p2A-GFP* KI, and *Stra8* $^{null\ GFP}$-KI ovaries (E14.5) by fluorescent sorting.
(b, c) The DDX4 (VENUS)-expressing cells were isolated from *Stra8* ^{wt}*-3FH-p2A-GFP* KI and *Stra8* $^{\Delta LXCXE/\Delta LXCXE}$ *-3FH-p2A-GFP* KI ovaries at E15.5 (B) and E18.8 (C) by fluorescent sorting.
(d) Summary table of the metrics for scRNA-seq analysis with STRA8 (GFP)-expressing (E14.5) and DDX4 (Venus)-expressing (E15.5 and E18.5) female germ cells. Indicated numbers of ovaries were pooled. The number of single cells that were subjected to RNA-seq analysis are shown before and after quality control. Purity of the isolated germ cells per total single cells that were subjected to RNA-seq analysis are shown.

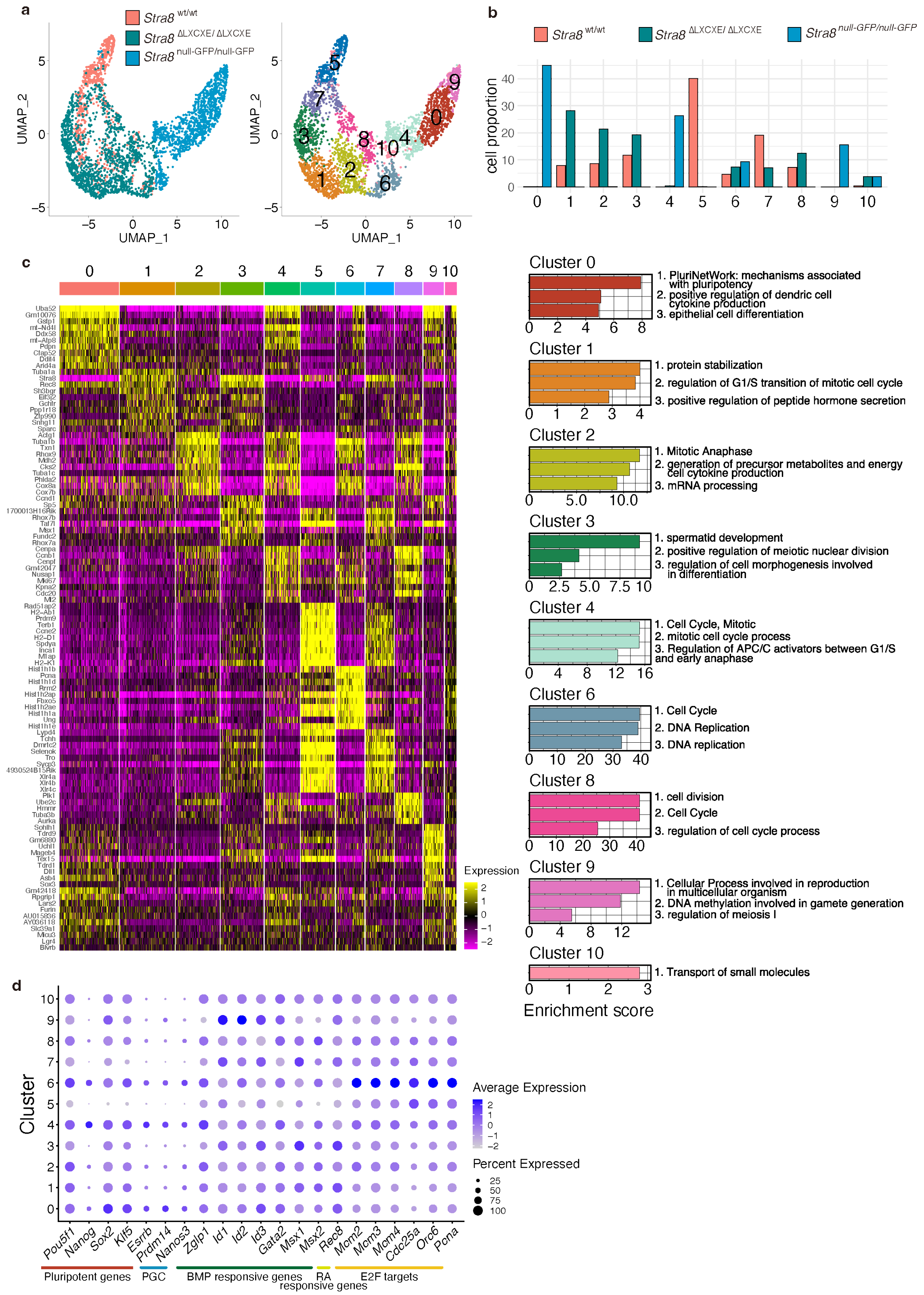

Supplementary Figure 5. DEGs in scRNA-seq analyses of E14.5 germ cells isolated from *Strab8* $^{wt/wt}$, *Strab8* $^{\Delta LXCXE/\Delta LXCXE}$ and *Strab8* $^{null\ GFP/null\ GFP}$ embryonic ovaries (Related to Figures 2, 3 and 4)

(a) UMAP representation of scRNA-seq transcriptomes for STRA8 (GFP)-expressing germ cells from *Stra8* $^{wt/wt}$-*3xFH-P2A-GFP*, *Stra8* $^{\Delta LXCXE/\Delta LXCXE}$ -*3xFH-P2A-GFP* and *Stra8* $^{null\ GFP/\ null\ GFP}$ knock-in ovaries at E14.5 (left). Clustering analysis of different gene expression patterns on UMAP-defined scRNA-seq transcriptomes of *Stra8* $^{wt/wt}$, *Stra8* $^{\Delta LXCXE/\Delta LXCXE}$ and *Stra8* $^{null\ GFP\ /\ null\ GFP}$ germ cells (right).
(b) Bar graph showing the proportion of *Stra8* $^{wt/wt}$, *Stra8* $^{\Delta LXCXE/\Delta LXCXE}$ and *Stra8* $^{null\ GFP\ /\ null\ GFP}$ germ cells among the clusters.
(c) Heat plot for the top 10 representative genes of each cluster (left). Gene enrichment analysis of DEGs in the UMAP-defined cell clusters (right).
(d) Dot plot representation of average scaled expression (color gradient) and the percentage of cells in each UMAP-defined cluster with detectable expression (dot size) for genes associated with BMP-signaling, RA-signaling and E2F-targets.

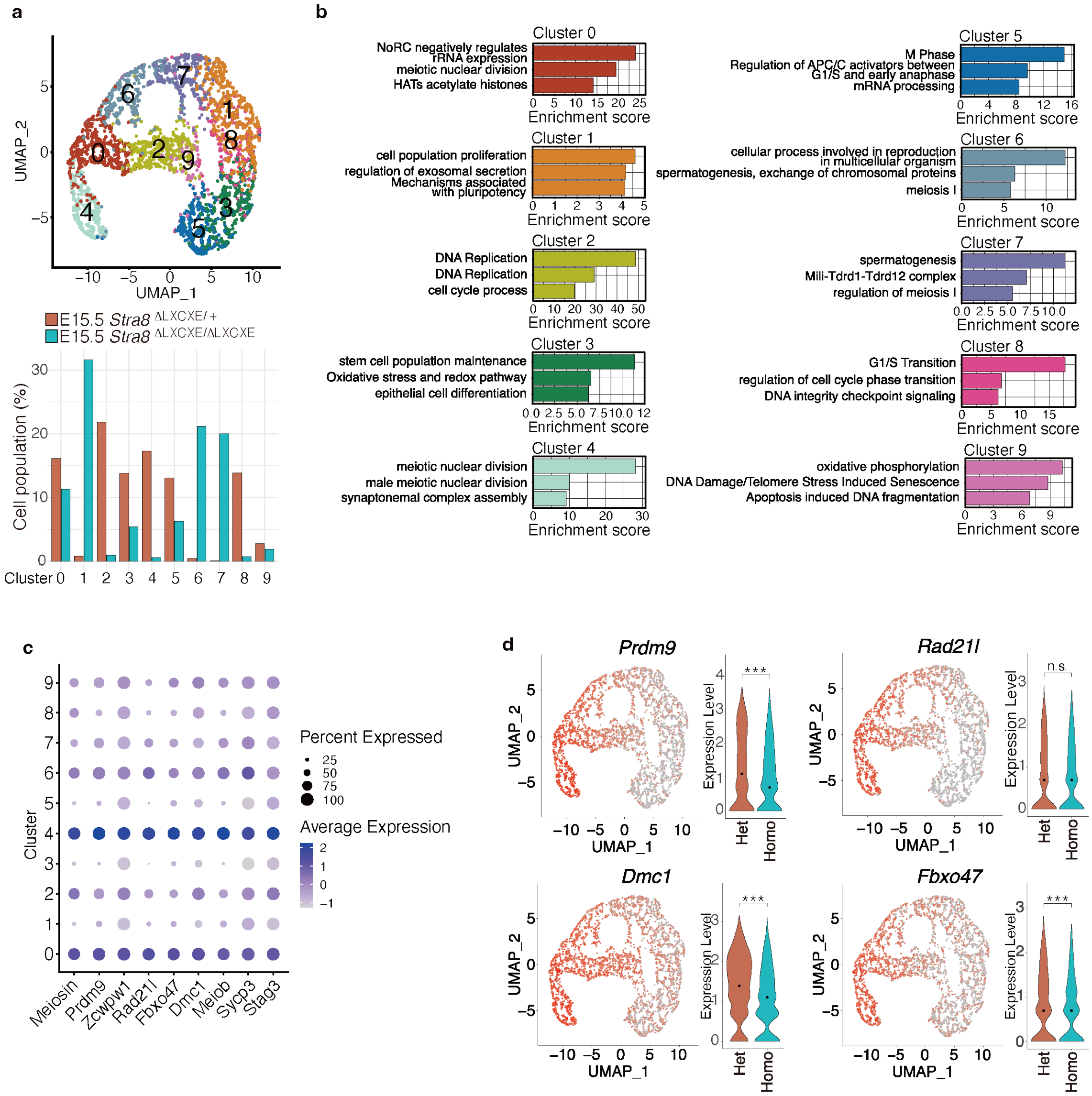

Supplementary Figure 6. Meiotic entry and subsequent progression of meiotic prophase were delayed in embryonic *Stra8*$^{\Delta LXCXE/\Delta LXCXE}$ oocytes (Related to Figure 5)

(a) Clustering analysis of different gene expression patterns on UMAP-defined scRNA-seq transcriptomes of the heterozygous control and homozygous *Stra8*$^{\Delta LXCXE/\Delta LXCXE}$ germ cells at E15.5 (top). Bar graph shows the proportion of the heterozygous control and homozygous *Stra8*$^{\Delta LXCXE/\Delta LXCXE}$ germ cells among the clusters (bottom).

(b) Gene enrichment-analysis of DEGs in the UMAP-defined clusters are shown. Top 3 biological processes ranked by enrichment score are indicated.

(c) Dot plot representation of average scaled expression (color gradient) and the percentage of cells in each UMAP-defined cluster with detectable expression (dot size) for genes associated with meiosis.

(d) Expression patterns and levels are shown on the UMAP plot and violin plot, respectively, for meiotic genes *Prdm9*, *Rad21L*, *Dmc1* and *Fbxo47*. ***: $p < 0.001$ (Wilcoxon rank-sum test).

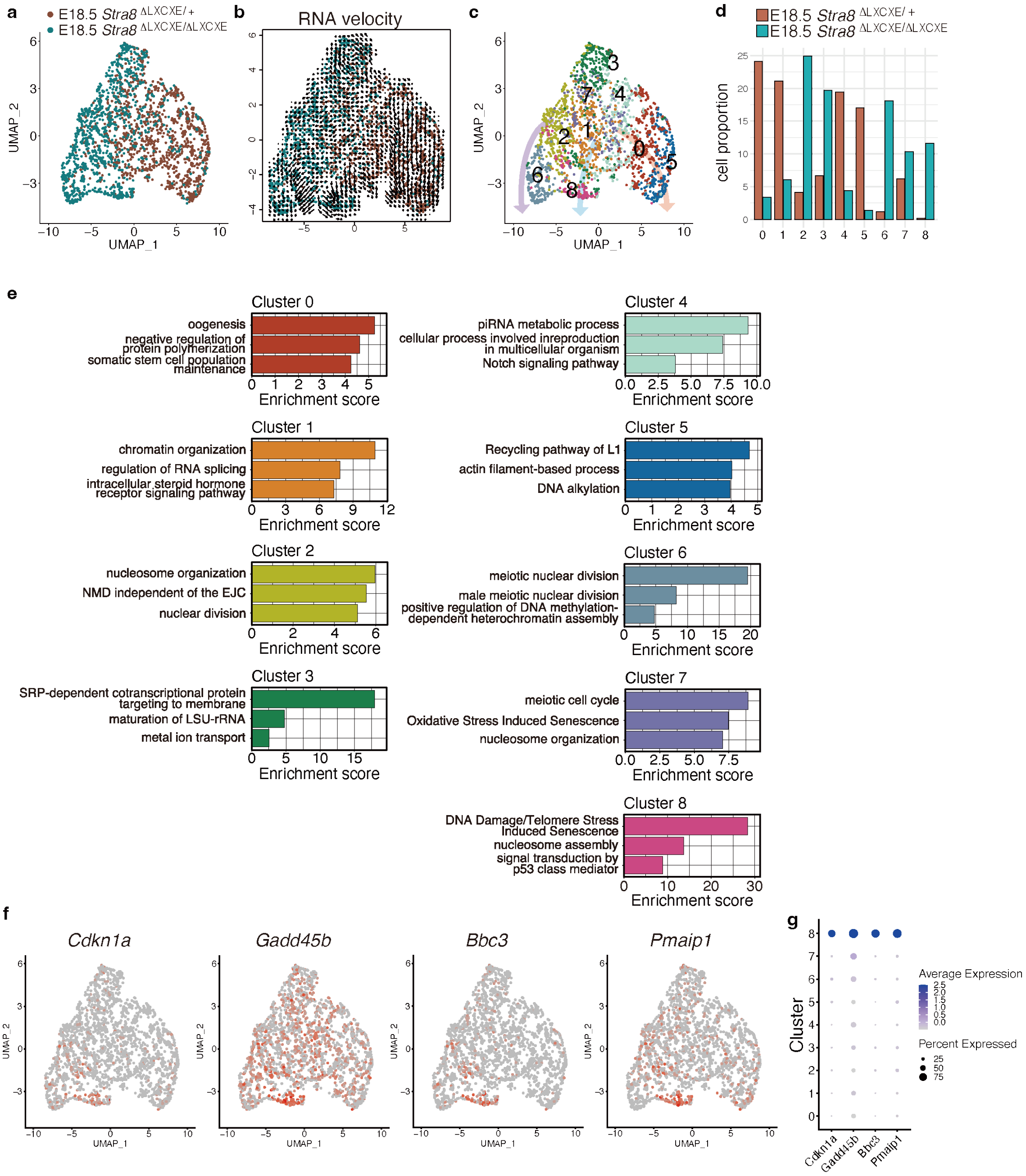

Supplementary Figure 7. DEGs in scRNA-seq analyses of E18.5 oocytes isolated from *Stra8*$^{\Delta LXCXE/+}$ and *Stra8*$^{\Delta LXCXE/\Delta LXCXE}$ embryonic ovaries (Related to Figure 6)

(a) Overlaid UMAP representation of scRNA-seq transcriptomes is shown for DDX4-expressing germ cells from heterozygous control and homozygous *Stra8*$^{\Delta LXCXE/\Delta LXCXE}$-*3xFH-P2A-GFP* knock-in ovaries at E18.5.

(b) The developmental trend of the individual cells was estimated by RNA velocity analysis. The velocities are visualized on the merged UMAP plot.

(c) Clustering analysis of different gene expression patterns on UMAP-defined scRNA-seq transcriptomes. Colored arrows indicate developmental directions estimated by RNA velocity.

(d) Bar graph shows the proportion of the heterozygous control and homozygous *Stra8*$^{\Delta LXCXE/\Delta LXCXE}$ germ cells among the clusters.

(e) Gene enrichment analysis of the DEGs at E18.5 in the UMAP-defined cell clusters.
(f) Expression patterns and levels are shown on the UMAP plot for the genes related to p53-dependent apoptosis/damage response (*Cdkn1a, Gadd45b, Bbc3,* and *Pmaip1*). ***: $p < 0.001$ (Wilcoxon rank-sum test).
(g) Expression patterns and levels are shown on the UMAP plot and violin plot, respectively for the genes related to p53-dependent apoptosis/damage response (*Cdkn1a, Gadd45b, Bbc3,* and *Pmaip1*). ***: $p < 0.001$ (Wilcoxon rank-sum test).

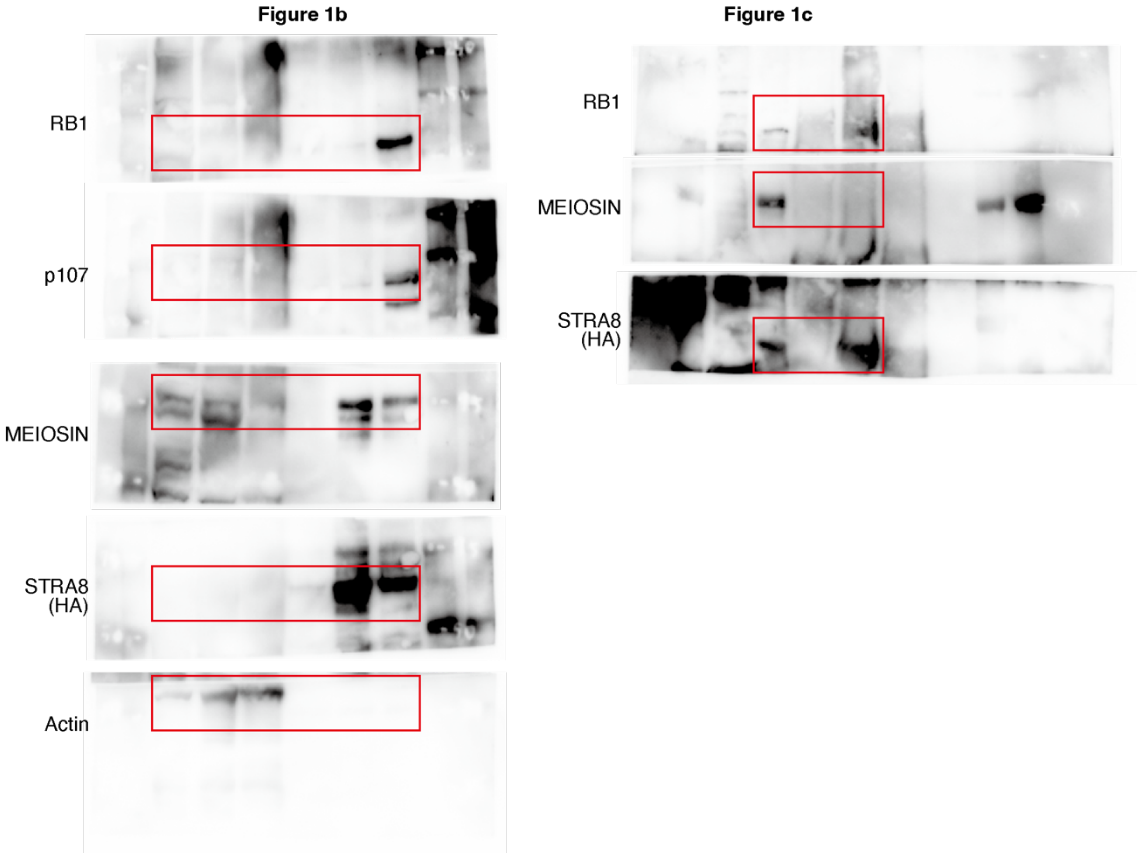

Supplementary Figure 8. Uncropped images of blots

For immunoblot of Figure1b and Figure1c, the input testis extracts and immunoprecipitates were run on the same gel. The immunoblot membrane was cut into separate pieces according to molecular weight marker, so that different proteins could be simultaneously probed with different antibodies.
