# Supplementary material for: STRA8–RB interaction is required for timely entry of meiosis in mouse female germ cells
Source: Nat Commun. 2023 Oct 25;14:6443. doi: 10.1038/s41467-023-42259-6 (PMC10600341; doi:10.1038/s41467-023-42259-6)
Supplement: Supplementary file 3 — Description of Additional Supplementary Files [file 41467_2023_42259_MOESM3_ESM.pdf]

Description of Additional Supplementary Files:

Supplementary Data 1: Gene enrichment analysis of highly expressed genes in STRA8-null cells

Tab 1 : The complete list of highly expressed genes in STRA8-null cells that was used for Gene enrichment analysis.

Tab 2 : The complete result of Gene enrichment analysis for the highly expressed genes in STRA8-null cells. The Description and LogP colonums of top 3 summary rows were used for Fig.2.

Supplementary Data 2: The list of DEGs in the clusters of scRNA-seq at E14.5.

Shown are the complete list of DEGs in the clusters of scRNA-seq at E14.5.

Supplementary Data 3: The reference gene set for the cell cycle estimation.

The genes that are defined to represent G1/S, S, G2, G2/M, and M/G1 are listed.

Supplementary Data 4: The list of DEGs in the clusters of scRNA-seq at E15.5.

Shown are the complete list of DEGs in the clusters of scRNA-seq at E15.5.

Supplementary Data 5: The list of DEGs in the clusters of scRNA-seq at E18.5.

Shown are the complete list of DEGs in the clusters of scRNA-seq at E18.5.

Supplementary Data 6: Primers and oligos used in this study.
