# Supplementary material for: STRA8–RB interaction is required for timely entry of meiosis in mouse female germ cells
Source: Nat Commun. 2023 Oct 25;14:6443. doi: 10.1038/s41467-023-42259-6 (PMC10600341; doi:10.1038/s41467-023-42259-6)
Supplement: Supplementary file 10 — Reporting Summary [file 41467_2023_42259_MOESM10_ESM.pdf]

nature research

Corresponding author(s): Kei-ichiro Ishiguro

Last updated by author(s): Jun 23, 2023

Reporting Summary

Nature Research wishes to improve the reproducibility of the work that we publish. This form provides structure for consistency and transparency in reporting. For further information on Nature Research policies, see our Editorial Policies and the Editorial Policy Checklist.

Statistics

For all statistical analyses, confirm that the following items are present in the figure legend, table legend, main text, or Methods section.

n/a	Confirmed	
☐	☒	The exact sample size (n) for each experimental group/condition, given as a discrete number and unit of measurement
☐	☒	A statement on whether measurements were taken from distinct samples or whether the same sample was measured repeatedly
☐	☒	The statistical test(s) used AND whether they are one- or two-sided<br>*Only common tests should be described solely by name; describe more complex techniques in the Methods section.*
☒	☐	A description of all covariates tested
☐	☒	A description of any assumptions or corrections, such as tests of normality and adjustment for multiple comparisons
☐	☒	A full description of the statistical parameters including central tendency (e.g. means) or other basic estimates (e.g. regression coefficient) AND variation (e.g. standard deviation) or associated estimates of uncertainty (e.g. confidence intervals)
☐	☒	For null hypothesis testing, the test statistic (e.g. F, t, r) with confidence intervals, effect sizes, degrees of freedom and P value noted<br>*Give P values as exact values whenever suitable.*
☒	☐	For Bayesian analysis, information on the choice of priors and Markov chain Monte Carlo settings
☒	☐	For hierarchical and complex designs, identification of the appropriate level for tests and full reporting of outcomes
☒	☐	Estimates of effect sizes (e.g. Cohen's d, Pearson's r), indicating how they were calculated

Our web collection on statistics for biologists contains articles on many of the points above.

Software and code

Policy information about availability of computer code

Data collection	no software was used for the data collection.
Data analysis	R (version 4.2.2), Microsoft Excel (version 16.48), SoftWoRx (ver.7.2.1, GE Healthcare)., Seurat packge for R (v.3.1.3), monocle (ver.2.14.0), RStudio (ver.2021.9.2.382), Bioworks (Ver. 3.3; Thermo Scientific), Xcalibur (Version 4.0, Thermo Fisher Scientific)

For manuscripts utilizing custom algorithms or software that are central to the research but not yet described in published literature, software must be made available to editors and reviewers. We strongly encourage code deposition in a community repository (e.g. GitHub). See the Nature Research guidelines for submitting code & software for further information.

Data

Policy information about availability of data

All manuscripts must include a data availability statement. This statement should provide the following information, where applicable:

- Accession codes, unique identifiers, or web links for publicly available datasets
- A list of figures that have associated raw data
- A description of any restrictions on data availability

All data supporting the conclusions are present in the paper and the supplementary materials. A reporting summary for this Article is available as Supplementary Information file. The source data (for Fig.2d, Fig. 2e, Fig. 2g, Fig. 3d, Fig. 3e, Fig.3f, Fig.4c, Fig.4d, Fig.4e,Fig. 5d, Fig. 5e, Fig. 5f, Fig. 5g, Fig. 6a, Fig. 6b, Fig. 6c, Fig. 7b, Fig. 7c, Fig. 8b, Fig.S2c, Fig.S3b, Fig.S5b, Fig.S6a, Fig.S7d) are provided with this paper. Sequencing data have been deposited in DDBJ Sequence Read Archive (DRA) under the accession DRA013182 for scRNA-seq data of E14.5 germ cells and DRA015395 for the scRNA-seq data of E15.5 and E18.5 germ cells.
Uncropped blots can be found in Supplementary Fig 8.
Reference genome for scRNA-seq, mm10 was obtained from 10x genomics website: https://support.10xgenomics.com/single-cell-gene-expression/software/release-notes/build#mm10_3.0.0

Original images can be obtain from Figshare: http://XXX or accession ?

Field-specific reporting

Please select the one below that is the best fit for your research. If you are not sure, read the appropriate sections before making your selection.

- [x] Life sciences
- [ ] Behavioural & social sciences
- [ ] Ecological, evolutionary & environmental sciences

For a reference copy of the document with all sections, see nature.com/documents/nr-reporting-summary-flat.pdf

Life sciences study design

All studies must disclose on these points even when the disclosure is negative.

Sample size	No statistical method was used to predetermine sample size. We followed the conventional way of quantification accepted in many of the published paper in meiosis research field and determined the sample size according to published papers (Horisawa-Takada, Y. et al. (2021) doi:https://doi.org/10.1038/s41467-021-23378-4, Ishiguro, K. I. et al. (2020) doi:10.1016/j.devcel.2020.01.010, Larose, H. et al., (2020) doi:10.1091/mbc.E20-05-0334.).
Data exclusions	No data was excluded.
Replication	Each conclusion in the manuscript was based on results that were reproduced in at least two independent experiments and in at least three independent mice of each genotype.
Randomization	Mice were categorized based on their genotypes. The genotypes were determined by PCR. For experiments other than those involving mice, samples were non-randomly chosen according to the genotype.
Blinding	The investigators were not blinded to allocation during the experiments or to outcome assessment.<br>This is because the phenotypes were quite obvious that observer can be sure without blind test.<br>Further, the observer unbiasedly and carefully performed the quantification with enough sample number to make sure the conclusion.

Reporting for specific materials, systems and methods

We require information from authors about some types of materials, experimental systems and methods used in many studies. Here, indicate whether each material, system or method listed is relevant to your study. If you are not sure if a list item applies to your research, read the appropriate section before selecting a response.

Materials & experimental systems

n/a	Involved in the study	
[ ]	[x]	Antibodies
[x]	[ ]	Eukaryotic cell lines
[x]	[ ]	Palaeontology and archaeology
[ ]	[x]	Animals and other organisms
[x]	[ ]	Human research participants
[x]	[ ]	Clinical data
[x]	[ ]	Dual use research of concern

Methods

n/a	Involved in the study	
[x]	[ ]	ChIP-seq
[x]	[ ]	Flow cytometry
[x]	[ ]	MRI-based neuroimaging

Antibodies

Antibodies used	The following antibodies were used for immunoblot (IB) and immunofluorescence (IF) studies: The following antibodies were used for immunoblot (IB) and immunofluorescence (IF) studies: rabbit anti-PLZF (IF, 1:1000, Abcam: ab189849), rabbit anti-DAZL (IF, 1:1000, ab34139), rabbit anti-HA (IB, IF, 1:1000, Abcam: ab9110) mouse anti-HA 12CA5 monoclonal Antibody (IF, 1:100, Roche: AB_514505), rabbit anti-H3S10P (IF, 1:2000, Abcam: ab5176), rabbit anti-SYCP1 (IF, 1:1000, Abcam ab15090), rabbit anti-DMC1 (IF, 1:500, Santa Cruz: SC-22768), mouse anti-MLH1 (IF, 1:500, BD Biosciences: 551092), rabbit anti-Actin (IB, 1:1000, Sigma A2066), rabbit anti-GFP (IF, 1:1000, ab6556), rat anti-TRA98 (IF, 1:1000, ab82527), rabbit anti-p107 (IB, 1:1000, Santa Cruz: SC-318), rabbit anti-FOXO3 (IF, 1:200, CST 2497), rat anti-NANOG (IF, 1:1000 Thermo: eBioMLC-51), rat anti-SYCP3 and guinea pig anti-SYCP3, rabbit and rat anti-STRA8, rabbit and gunia pig anti-MEIOSIN N-terminal (a.a. 1-224) and rabbit and gunia pig anti-MEIOSIN C-terminal (a.a. 405-589) as described previously (Ishiguro et al. 2020),<br>gunia pig STRA8 (IF, 1:1000, our home made in this study), rabbit RB1-C terminal (a.a. 479-921)(IF, 1:1000, our home made in this study).<br>Following secondary antibodies were used : Donkey anti-rat IgG-Alexa Fluour 488 (IF, 1:1000, Thermo Fisher, A21208), Donkey anti-rabbit IgG-Alexa Fluour 488 (IF, 1:1000, Thermo Fisher, A21206), Donkey anti-rabbit IgG-Alexa Fluour 555 (IF, 1:1000, Thermo Fisher, A31572),Goat anti-rat IgG-Alexa Fluour 647 (IF, 1:1000, Thermo Fisher, A21247), , Donkey anti-mouse IgG-Alexa Fluour 647 (IF,

1:1000, Thermo Fisher, A31571),Goat anti-Gunia pig IgG-Alexa Flouur 555 (IF, 1:1000, Abcam ab150186), Donkey Anti-Mouse IgG Alexa 488 (IF, 1:1,000, Thermo Fisher, A21202), Donkey Anti-Mouse IgG Alexa 555 (IF, 1:1,000, Thermo Fisher, A31570), Goat Anti-Guinea pig IgG Alexa 647 (IF, 1:1,000, Thermo Fisher, A21450), Goat Anti-Rabbit IgG Alexa 647 (IF, 1:1,000, Thermo Fisher, A21245), Goat anti-rabbit IgG-Alexa Fluour 647 (IF, 1:1000, Thermo Fisher, A21244) Donkey anti-rabbit IgG-Alexa Fluour 647 (IF, 1:1000, Thermo Fisher, A31573), Donkey anti-rat IgG-Alexa Fluour 555 (IF, 1:1000, Thermo Fisher, A48270), Donkey anti-rabbit IgG-Alexa Fluour 568 (IF, 1:1000, Thermo Fisher, A10042), Goat anti-rat IgG-Alexa Fluour 568 (IF, 1:1000, Thermo Fisher, A11077), Goat anti-rabbit IgG-Alexa Fluour 568 (IF, 1:1000, Thermo Fisher, A11011), Donkey anti-rabbit IgG-Alexa Fluour 488 (IF, 1:1000, Thermo Fisher, A21206), , Goat anti-Gunia pig IgG-Alexa Fluour 488 (IF, 1:1000, Abcam ab150185), Goat anti-Gunia pig IgG-Alexa Fluour 647 (IF, 1:1000, Abcam ab150187), Anti-Rabbit IgG HRP (IB, 1:5000, Cytiva GE, NA934), Anti-Mouse IgG HRPIB, 1:5000, Cytiva GE, NA931), Anti-Guinea pig IgG HRP (IB, 1:2000, Abcam, ab6908)

Validation

The newly generated antibodies in this study were validated by western blotting and immunostaining .
The following our home made antibodies were validated for immunofluorescence (IF) in our previous studies: rat anti-SYCP3 (IF, 1:1000, our home made), gunia pig anti-SYCP3 (IF, 1:2000, our home made) , rat anti-STRA8(IF, 1:1000, our home made), Rabbit anti-STRA8 (IB, IF, 1:1,000, our home made), Rabbit anti-MEIOSIN-N (IB, IF, 1:1,000, our home made), Guinea pig anti MEIOSIN-N (IB, IF, 1:1,000, our home made) .
The following antibodies were validated for immunoblot (IB) and immunofluorescence (IF) in manufacture's website :
Rabbit anti-HA (IB, IF, 1:1,000, Abcam, ab9110), Rabbit anti-HA (IB, IF, 1:1,000, Roche, 11583816001), Rabbit anti-PLZF (IF, 1:1,000, Abcam, ab189849), Rabbit anti-DAZL (IF, 1:1,000, Abcam, ab34139), Rabbit anti-H3S10P (IF, 1:1,000, Abcam, ab5176), Rabbit anti-SYCP1 (IF, 1:1,000, Abcam, ab15090), rabbit anti-DMC1 (IF, 1:500, Santa Cruz: SC-22768), mouse anti-MLH1 (IF, 1:500, BD Biosciences: 551092), rabbit anti-Actin (IB, 1:2000, sigma A2066), Rabbit anti-GFP (IF, 1:1,000, Abcam, ab6556), Rat anti-TRA98 (IF, 1:1,000, Abcam, ab82527), Rabbit anti-p107 (IB, 1:1,000, Santa Cruz, SC-318), Rabbit anti-FOXO3 (IF, 1:200, Cell Signaling Technology, 2497), Rat anti-NANOG (IF, 1:1,000, Thermo Fisher Scientific, 14-5761-80)

Animals and other organisms

Policy information about studies involving animals; ARRIVE guidelines recommended for reporting animal research

Laboratory animals

LXCXE mutant Stra8-3xFLAG-HA-p2A-GFP knock-in (Stra8 dLXCXE-3FH KI) and Stra8-null GFP knock-in (Stra8 null GFP-KI) mouse lines were generated in this study. Stra8 dLXCXE-3FH KI and Stra8 null GFP-KI mice were C57BL/6 background. WT Stra8-3xFLAG-HA-p2A-GFP knock-in (Stra8-3FH KI) was generated in previous study (Ishiguro et al 2020).Mvh/Ddx4-Venus transgenic mice : B6D2-Tg (Ddx4-Venus) 1Rbrc (Shiura et al. 2013) were used for fluorescent sorting of female germ cells.
Whenever possible, each knockout animal was compared to littermates or age-matched non-littermates from the same colony, unless otherwise described. We used E14-18, P0, P2, P17, 4w and 8w female animals for histological analysis and expression analysis, E14, E15, E18 female embryos for scRNA-seq and chromosome spread IF experiments, otherwise indicated in the figure legends.
Male mice were used for immunoprecipitation of testis extracts (age : postnatal day 10-12 old), histological analysis of testes, and immunostaining of testes (age : postnatal day 8, 4- weeks and 8-weeks old). Both males and females were used for Stra8-3FH KI, Stra8 dLXCXE-3FH KI and Stra8 null GFP-KI mice, Mvh/Ddx4-Venus transgenic mice .
Housing conditions for the mice were under 12 hours dark/12 hours light cycle, ambient temperature at 20-23 degree C and humidity 40-60 % .
Antibody production using rabbit and guinia pig was done by a contractor (Kiwa Laboratory Animals Co., Ltd.). The handling of these animals was carried out according the contractor's animal experimental protocols.

Wild animals

No wild animal was used.

Field-collected samples

No field-collected samples were used in the study.

Ethics oversight

Animal experiments were approved by the Institutional Animal Ethics Committees of Kumamoto University (approval F28-078, A2020-006, A2022-01, A28-026, A30-001).

Note that full information on the approval of the study protocol must also be provided in the manuscript.
